# Adamantane-Based Micro- and Ultra-Microporous Frameworks for Efficient Small Gas and Toxic Organic Vapor Adsorption

**DOI:** 10.3390/polym11030486

**Published:** 2019-03-13

**Authors:** Wenzhao Jiang, Hangbo Yue, Peter S. Shuttleworth, Pengbo Xie, Shanji Li, Jianwei Guo

**Affiliations:** 1 School of Chemical Engineering & Light Industry, Guangdong University of Technology, Guangzhou 510006, China; jwz.max@foxmail.com; 2 Department of Polymer Physics, Elastomers and Energy, Institute of Polymer Science and Technology, CSIC, 28006 Madrid, Spain; peter@ictp.csic.es; 3 Guangzhou Institute of Technology, Guangzhou 510075, China; xpbty@21cn.com (P.X.); hnlsj2004@163.com (S.L.)

**Keywords:** organic framework, ultra-microporosity, adsorption, adamantane, Sonogashira-Hagihara coupling

## Abstract

Microporous organic polymers and related porous materials have been applied in a wide range of practical applications such as adsorption, catalysis, adsorption, and sensing fields. However, some limitations, like wide pore size distribution, may limit their further applications, especially for adsorption. Here, micro- and ultra-microporous frameworks (HBPBA-D and TBBPA-D) were designed and synthesized via Sonogashira–Hagihara coupling of six/eight-arm bromophenyl adamantane-based “knots” and alkynes-type “rod” monomers. The BET surface area and pore size distribution of these frameworks were in the region of 395–488 m^2^ g^−1^, 0.9–1.1 and 0.42 nm, respectively. The as-made prepared frameworks also showed good chemical ability and high thermal stability up to 350 °C, and at 800 °C only 30% mass loss was observed. Their adsorption capacities for small gas molecules such as CO_2_ and CH_4_ was 8.9–9.0 wt % and 1.43–1.63 wt % at 273 K/1 bar, and for the toxic organic vapors n-hexane and benzene, 104–172 mg g^−1^ and 144–272 mg g^−1^ at 298 K/0.8 bar, respectively. These are comparable to many porous polymers with higher BET specific surface areas or after functionalization. These properties make the resulting frameworks efficient absorbent alternatives for small gas or toxic vapor capture, especially in harsh environments.

## 1. Introduction

In recent years, the design and construction of microporous organic polymers (MOPs) [1] has attracted interest from both the scientific community and industry at large due to their huge potential in applications such as gas storage/separation, catalysis, sensors, and drug delivery [2,3,4,5]. Different kinds of MOPs have been developed, such as covalent organic frameworks [6], hypercross-linked polymers [7], polymers of intrinsic microporosity [8], conjugated microporous polymers [9], covalent triazene frameworks [10], etc. These materials have been reported as the most promising materials for carbon dioxide capture and storage owing to their low mass density, large specific surface area, and high thermal and chemical stability. For instance, COF-103 exhibited a CO_2_ capacity uptake of 7.6 wt % at 298 K/55 bar [11]. The microporous polymer PBI-Ads [12] and network A [13] also showed high CO_2_ adsorption capacities of 17.3 wt % at 273 K/1 bar and 11.7 wt % at 273 K/1 bar, respectively.

Intense research efforts have been devoted to controlling the surface area and porosity of MOPs, including altering the reaction conditions [14] or introducing suitable templates [15]. It is worthy to note that characteristics of MOPs are affected not only by the synthesis method, but also by the choice of the building blocks used. In some cases, the pore size and surface areas of MOPs are significantly influenced by the geometry of the building blocks utilized to synthesize the frameworks [16]. MOPs made from tetrahedral units, such as tetraphenylmethane, tetraphenylsilane, adamantane and its derivatives, have demonstrated very high specific surface areas. For instance, tetra(4-(dihydroxy)borylphenyl)-methane based COF-102 and tetra(4-(dihydroxy)borylphenyl)silane based COF-103 exhibited BET surface areas up to 3530–3620 m^2^ g^−1^ [11]. Conjugated microporous polymers PPN-3 and PPN-4 prepared by self-condensation of tetrakis(4-bromophenyl)admantane and tetrakis(4-bromophenyl)silane showed exceptionally high BET surface areas of 4221 and 6461 m^2^ g^−1^, respectively [17]. Owing to their rigid three-dimensional skeleton and high physicochemical stability, adamantane and its derivatives have been utilized as building blocks for the construction of porous frameworks, and the resulting MOPs also displayed high thermal and chemical stability. For instance, Chang et al. [18,19] synthesized a clickable microporous polymer based on 1,3,5,7-tetrakis(4-iodopheny)adamantane with high surface area (442–665 m^2^ g^−1^) and good thermo-stability (340 °C) and a series of post-functionalized polymers with adamantane core, which showed good CO_2_ uptake capacities (880–1890 m^2^ g^−1^, 27–39 cm^3^ g^−1^ at 298 K/1 bar). MOPs-Ad [20] and HBPBAs-Ad [21] constructed from tetrakis(4-bromophenyl)admantane and hexaphenylbiadamantane, showed high thermal (stable up to >500 °C) and chemical (strong acid and base) stability. Adamantane-based SOF polymers demonstrated a residual weight of 60–70 wt % at 800 °C. Furthermore, the SOF polymers demonstrated good gas and toxic organic vapor adsorption capabilities: 5.2–15.1 wt % of CO_2_ (273 K/1 bar), 1.0–2.4 wt % of CH_4_ (273 K/1 bar), 279–407 mg g^−1^ of benzene, and 195–250 mg g^−1^ of n-hexane (298 K/0.8 bar) [22]. However, these adamantane-based MOPs were reported to be amorphous, having a wide pore size distribution, and relatively low surface area, which may limit their future applications in gas storage and toxic vapor uptakes. Therefore, in recent years the scientific focus has been mainly concentrated to on MOPs with ultra-microporosity (pore widths <0.7 nm). Previous studies have shown that efficient and high gas uptake can be achieved in these ultra-microporous frameworks. For example, a uniformly robust three-dimensional cage-like ultra-microporous network (S_BET_ = 2247 m^2^ g^−1^) prepared from condensation of triptycene-based hexamine (THA) and hexaketocyclohexane (HKH) octahydrat. The result showed that the presence of periodic nitrogen atoms, aromatic phenyl, and pyrazine rings in the network were useful for the gas adsorption (CO_2_, 26.7 wt % and CH_4_, 2.4 wt % at 273 K/1 bar) [23]. Wang et al. [24] reported ultramicroporous semi-cycloaliphatic polyimides (S_BET_ = 900–1108 m^2^ g^−1^) to reduce the charge-transfer (CT) interactions between the neighboring polyimide segments and improve adsorption capacities for gas and organic vapor (CO_2_, 23.75 wt % at 273 K/1 bar; benzene, 176 wt % at 298 K/0.9 bar). To the best of our knowledge, most of the reported ultra-microporous frameworks contain reversibly formed bonds from heteroatoms such as N, O, etc., rather than being solely of a C–C covalent bonding construction [23,24]. However, adamantane-based ultra-microporous framework with solely of a C–C covalent bonding construction is rarely reported. Hence, it is necessary to develop a facile method to prepare rigid adamantane-based ultra-microporous framework that possesses excellent thermal stability and high gas/organic vapor adsorption capacity.

According to the Marissen’s theory [25], macroscopic mechanical principles from structural engineering can be applied to the design of molecular rigid structures, such as, recently reported adamantane-based SOF polymers [22]. Herein, six/eight-arm bromophenyl adamantane-based “knot” and alkynes-type “rod” monomers were connected using a Sonogashira–Hagihara coupling reaction to generate two porous organic frameworks, HBPBA-D and TBBPA-D. In addition, their topological structures, porosity parameters, as well as their adsorption capacities for small gas molecules (CO_2_ and CH_4_) and toxic organic vapors (n-hexane and benzene) were investigated. It was also found that their synthesized frameworks exhibited good thermal and chemical stability, a narrow pore size distribution (0.9–1.1 and 0.42 nm, respectively), and were comparable to state-of-the-art MOPs in terms of gas and vapor capture, making them potential candidates to addressing current Global Warming and air quality issues.

## 2. Materials and Methods

### 2.1. Materials

Chemicals including triethylamine (TEA), 1-methyl-2-pyrrolidinone (NMP), tetrahydrofuran (THF), dimethyl formamidine (DMF), chloroform, methanol, acetone, Pd(PPh_3_)_4_, CuI, and 1,4-diethynylbenzene were purchased from commercial suppliers (Shanghai Macklin Biochemical Co., Ltd., Shanghai, China) and used without further purification. 3,3′,5,5′,7,7′-hexakis(4-bromophenyl)-1,1′-biadamantane (HBPBA) and 1,3,5,7-tetrakis(1,3-bibromophenyl) adamantane (TBBPA) were synthesized according to the reported procedures [22].

### 2.2. Measurements

Solid-state cross polarization magic angle spinning ^13^C CP/MAS NMR experiments were recorded on a Bruker Avance III HD 400 spectrometer. Elemental analysis was recorded on a Perkin Elmer Series II 2400 analyzer. Scanning electron microscopy (SEM, Hitachi, Tokyo, Japan) images were performed on Hitachi S-4800 with an acceleration voltage of 6.0 KV. Thermogravimetric analysis (TGA) data were recorded with NETZSCH STA 409 PC thermal analyzer system (NETZSCH, Freistaat Bayern, Germany) in the temperature range 30–800 °C at a heating rate of 10 °C/min under N_2_ atmosphere. X-ray diffraction (XRD) patterns of the samples were acquired from 5° to 90° by Bruker D8 X-ray diffraction instrument (Bruker, Rheinstetten, Germany). The nitrogen and carbon dioxide adsorption–desorption isotherms were measured on a 3H-2000PM2 analyzer (Beishide Instrument Technology (Beijing) Co., Ltd., Beijing, China), and the adsorption of carbon dioxide and methane was measured on 3H-2000PS2 (Beishide Instrument Technology (Beijing) Co., Ltd., Beijing, China) apparatus at 273 K/1 bar. The benzene and n-hexane molecule adsorption isotherms were measured at 298 K/0.8 bar using a 3H-2000 PW (Beishide Instrument Technology (Beijing) Co., Ltd., Beijing, China) analyzer.

### 2.3. Synthesis of HBPBA-D and TBBPA-D

HBPBA (300 mg, 0.25 mmol), 1,4-diethynylbenzene (113.6 mg, 0.9 mmol), Pd(PPh_3_)_4_ (47.66 mg, 15 mol%), CuI (10.47 mg, 20 mol%), and 100 mL of TEA/NMP (50 mL/50 mL) were added into a 250 mL Schlenk flask and degassed by three freeze–thaw cycles. The mixture was then heated to 100 °C for 24 h under Argon atmosphere. The crude product was filtered and washed with methanol and THF. The insoluble powder was extracted with THF in Soxhlet apparatus for 24 h and dried under vacuum to obtain HBPBA-D (279 mg, 90% yield). Anal. Calcd for C_104_H_72_: C, 94.54; H, 5.46. Found: C, 93.56; H, 5.76. Using a similar protocol, TBBPA-D (264 mg, 88% yield) was synthesized from 1,3,5,7-tetrakis(1,3-bibromophenyl)adamantane (TBBPA) as a “rod” monomer. Anal. Calcd for C_80_H_54_: C, 94.67; H, 5.33. Found: C, 92.67; H, 5.47.

## 3. Results and Discussion

### 3.1. Synthesis and Characterization of HBPBA-D and TBBPA-D

**Figure 1:**
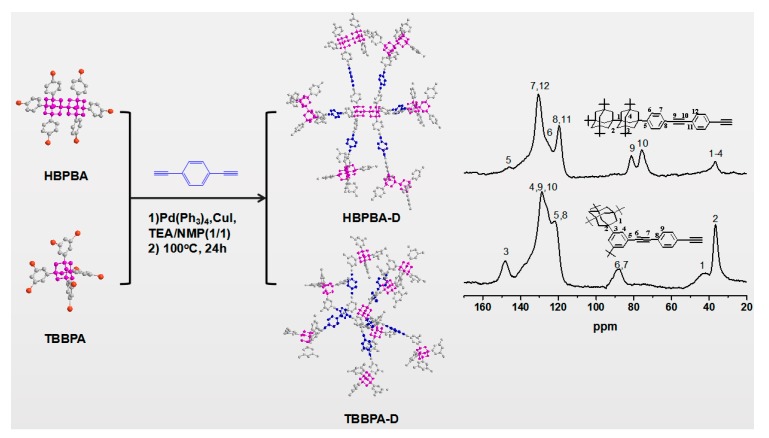
Synthetic route to HBPBA-D and TBBPA-D and ^13^C CP/MAS NMR spectra.

As shown in Figure 1, HBPBA-D and TBBPA-D were synthesized via Sonogashira–Hagihara coupling reaction. This synthetic approach provides a relatively simple way of producing the desired frameworks with high yields (about 90%). The resulting frameworks were characterized by ^13^C CP/MAS NMR (Figure 1). In the ^13^C NMR spectra, the peaks at regions around 30.0–45.0 ppm are ascribed to the resonance of adamantane moieties. The appearance of peaks at around 120–130 ppm are attributed to unsubstituted phenyl carbons. In addition, the resonance peaks near from 77 to 87 ppm were observed due to the carbon in the alkynyl linker. Detailed assignment of the resonances for particular carbon types in each compound is presented as follows: HBPBA-D (^13^C CP-MAS NMR) δ: 149.2 ppm (C5), 130.9 ppm (C7, C12), 124.5 ppm (C6), 120.8 ppm (C11, C8), 77–83 ppm (C9, C10), 40.2–35.3 (C1–C4). TBBPA-D (^13^C CP-MAS NMR) δ: 148.1 ppm (C3), 128.6 ppm (C4, C9, C10), 121.7 ppm (C5, C8), 87 ppm (C6, C7), 36.7–42 ppm (C1, C2).

**Figure 2:**
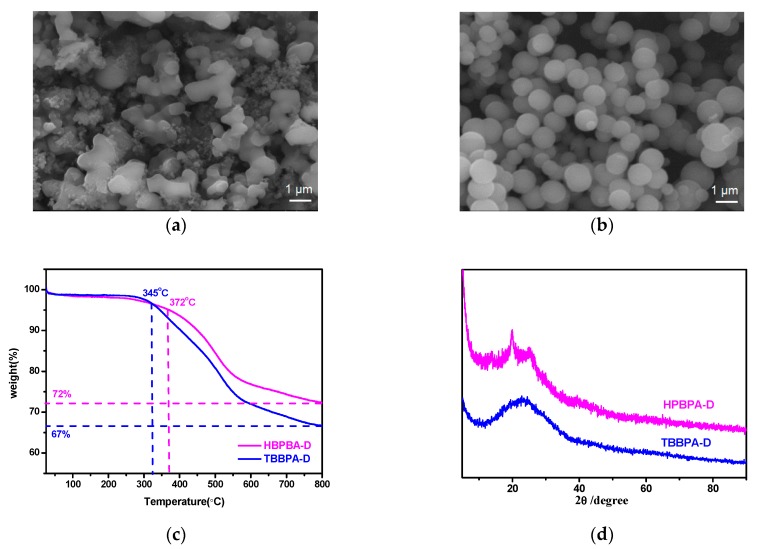
SEM images of (**a**) HBPBA-D and (**b**) TBBPA-D; (**c**) TGA plots of HBPBA-D and TBBPA-D; (**d**) XRD pattern of HBPBA-D and TBBPA-D.

SEM images (Figure 2a,b) show that HBPBA-D is composed of agglomerates of irregular shape particles, while TBBPA-D consists of relatively uniform mono-dispersed solid microspheres. The TGA curves (Figure 2c) show that HBPBA-D and TBBPA-D were stable up to 345–372 °C prior to decomposition. The residual weight of the two materials at 800 °C accounted for 67–72 wt % of their original weight, which is superior to many adamantane-based microporous polymers [26,27]. All the products were insoluble in strong acidic/basic solutions as well as many organic solvents such as THF, DMF, chloroform, and acetone. The broad features in the XRD spectra (Figure 2d) suggest that both frameworks are amorphous, owing to the likely irreversible reactions and framework interpenetration [28].

### 3.2. Porosity of HBPBA-D and TBBPA-D

**Figure 3:**
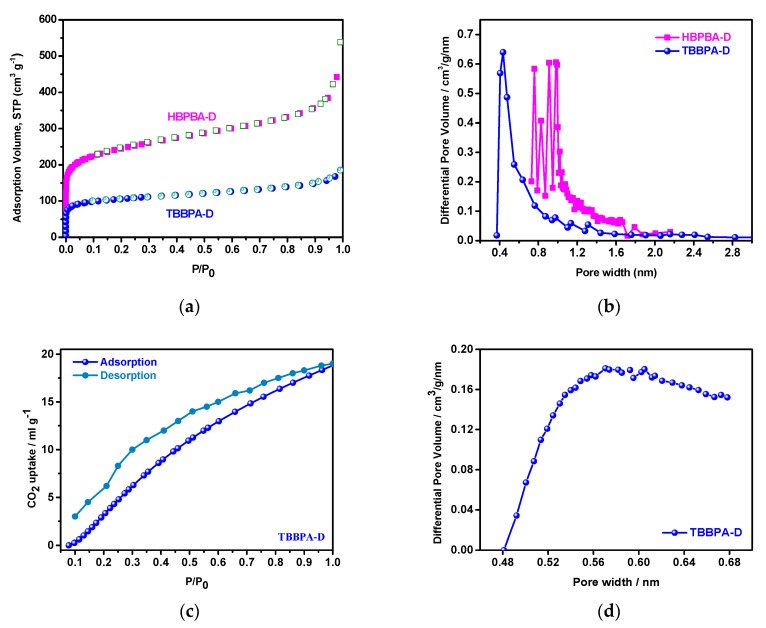
N_2_ sorption isotherms of (**a**) HBPBA-D and TBBPA-D at 77 K/1 bar; (**b**) PSD profile calculated by NLDFT; (**c**) CO_2_ sorption isotherms of TBBPA-D at 273 K; (**d**) PSD of TBBPA-D calculated using the H-K method.

The porosities of HBPBA-D and TBBPA-D were investigated by nitrogen sorption analysis at 77 K/1 bar with corresponding porosity results shown in Figure 3 and summarized in Table 1. In Figure 3a, HBPBA-D and TBBPA-D showed type I isotherms character with a steep rise at low relative pressure range (P/P_0_ < 0.01), indicating them to be microporous with an apparent BET surface area of 488 m^2^ g^−1^ and 395 m^2^ g^−1^, respectively. The pore size distributions (PSD) of the samples calculated using nonlocal density functional theory (NLDFT) showed HBPBA-D to have pore sizes in the range of 0.92–1.1 nm, and TBBPA-D to have a uniform pore size of 0.42 nm, and to be ultra-microporous. In this work, both HBPBA and TBBPA are rigid multifunctional building blocks, but their geometrical sizes and shapes are different. Compared with TBBPA, the larger building block of HBPBA can increase the pore dimensions. It is also found that the cross-linking density in the frameworks can affect the pore size and specific surface area of resultant the resultant materials. HBPBA has six p-bromophenyl arms stretching from two-linked adamantanes cores, but TBBPA has four m-dibromophenyl in one adamantane core. The presence of the higher density of rigid building blocks facilitates the sub-division of the space into smaller pores by framework segmentation.

To probe the ultra-microporosity of TBBPA-D, carbon dioxide was used due to its higher kinetic energy compared to nitrogen and thus better and quicker narrow pore penetrations. Figure 3c shows a typical type III carbon dioxide adsorption isotherm, indicating the behavior of permanent microporosity. Its PSD was estimated by H-K method from the adsorption of the carbon dioxide isotherm, showing a main peak at 0.59 nm (Figure 3d), which is in the ultra-micropore (<0.7 nm) range.

### 3.3. Small Gas and Toxic Organic Vapor Adsorption

**Figure 4:**
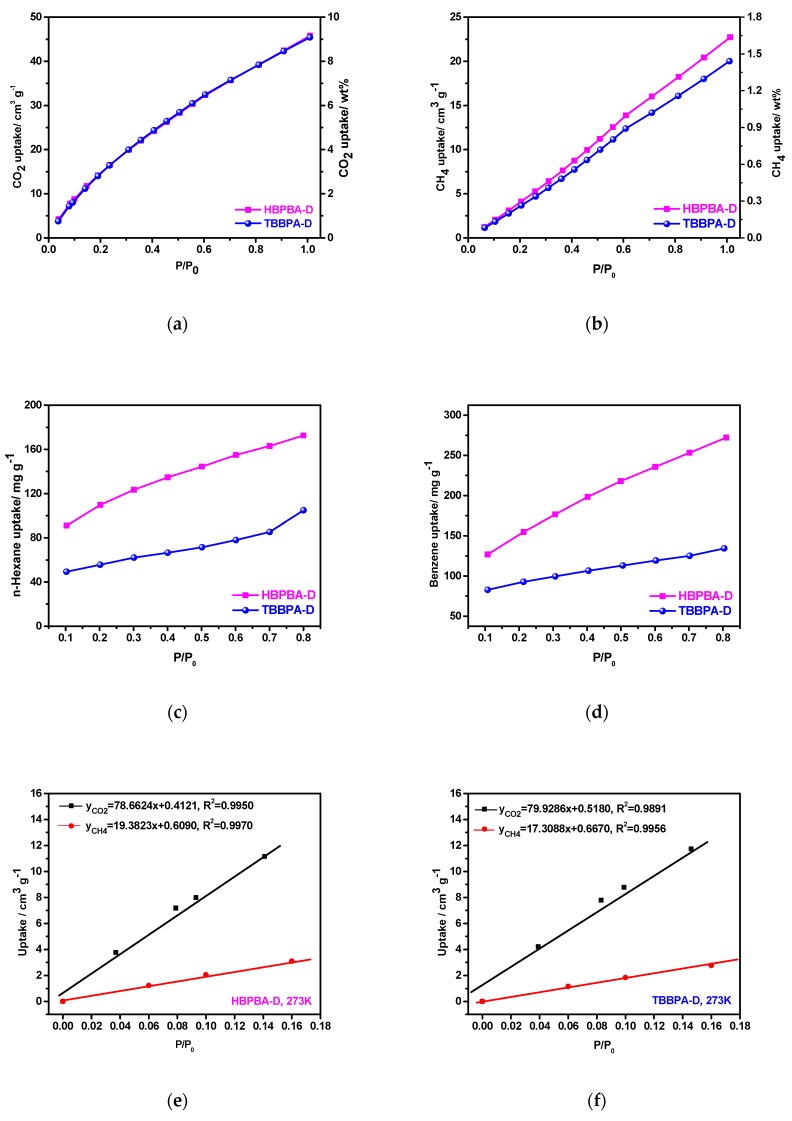
Adsorption isotherms of (**a**) CO_2_ at 273 K/1 bar, (**b**) CH_4_ at 273 K/1 bar (**c**) n-hexane, and (**d**) benzene at 298 K/0.8 bar; Selectivity calculation of CO_2_/CH_4_ for the (**e**) HBPBA-D and (**f**) TBBPA-D according to the Henry’s law at 273 K.

The small gas (CO_2_ and CH_4_) adsorption performance of HBPBA-D and TBBPA-D were measured at 273 K/1 bar. As shown in Figure 4a, despite having much lower surface areas than competitor microporous polymers with higher BET specific surface under the same condition, such as PAF-1(9.1 wt %, S_BET_ = 5640 m^2^ g^−1^) [29], HTPs (5–10.3 wt %, S_BET_ = 569–914 m^2^ g^−1^) [30], commercially available BPL carbon(9.2 wt %) [13], COP-3C (9.24 wt %, S_BET_ = 940 m^2^ g^−1^) [31] and MOPs-Ad (5.2–10.3 wt %, S_BET_ = 282–974 m^2^ g^−1^) [20], HBPBA-D and TBBPA-D can absorb 9.0 wt % and 8.92 wt % of CO_2_, respectively at 273 K/1 bar. The CH_4_ uptake capacities of the two frameworks at 273 K/1 bar were also studied, and the results presented in Figure 4b. The CH_4_ uptake capacities of HBPBA-D and TBBPA-D (1.63 and 1.43 wt %, respectively) are again higher than that of many reported porous polymers at 273 K/1 bar, such as POP (1.04–1.45 wt %, S_BET_ = 791–983 m^2^ g^−1^) [32], adamantane-based NOPs (0.78–1.32 wt %, S_BET_ = 526–1178 m^2^ g^−1^) [27] and HBPBAs-Ad (1.4–1.8 wt %, S_BET_ = 742–891 m^2^ g^−1^) [21], PPF (1–1.32 wt %, S_BET_ = 1740 m^2^ g^−1^) [33] and PCN (0.91–1.66 wt %, S_BET_ = 393–721 m^2^ g^−1^) [34]. According to Henry’s law, the CO_2_/CH_4_ selectivity of HBPBA-D and TBBPA-D at 273 K and pressure of less than 0.18 bar was calculated to be 4.1 and 4.6, respectively (Figure 4e,f), which is superior to many other polymers under the same testing conditions, such as CP-CMPs (3.4–4.2 at 273 K) [35], TEPS-TPA (3.9 at 273 K) [36] and triphenylamine-containing MOPs (3.4–4.3 at 273 K) [37]. Compared to the prepared HBPBA-D framework, TBBPA-D possesses a narrower pore size distribution (0.42–0.59 nm), which is advantageous for small molecule adsorption and thus [20], displaying a higher CO_2_/CH_4_ selectivity. The CO_2_ and CH_4_ adsorption at 273 K/1 bar could be dependent on the pore size and BET surface areas. Although TBBPA-D has lower BET surface area than HBPBA-D, the former showed compared or better small gas adsorptioin or CO_2_/CH_4_ selectivity abilities. The previous study [20] has revealed that ultra-micropores were adavantageous for the small gas adsorption and selectivity.

HBPBA-D and TBBPA-D were also tested for use in the adsorption of n-hexane and benzene vapors at 298 K/0.8 bar, with the results shown in Figure 4c,d. Compared to many other MOP materials under the same testing environment, such as conjugated microporous (120–272 mg g^−1^, S_BET_ = 309–1030 m^2^ g^−1^) [38], PAN (200–210 mg g^−1^, S_BET_ = 925–1242 m^2^ g^−1^) [39], PAF-S (262.96 mg g^−1^, S_BET_ = 1503 m^2^ g^−1^) [40], and SOFs (159.2 mg g^−1^, S_BET_ = 332–1049 m^2^ g^−1^) [22], HBPBA-D presented one of the highest adsorption capacities reported for benzene, 272.3 mg g^−1^. In addition, both HBPBA-D and TBBPA-D frameworks showed higher adsorption benzene vapor adsorption capacities compared to that for the n-hexane vapor. This difference is most likely due to the generated π-π interactions between the two frameworks and the benzene molecules [22]. When the total vapor uptake capacity of the two frameworks are compared, i.e., 172.5 mg g^−1^ and 104.8 mg g^−1^ of n-hexane and 272.3 mg g^−1^ and 144.3 mg g^−1^ of benzene for HBPBA-D and TBBPA-D respectively, it is likely that the molecule size of n-hexane (0.72 nm) and benzene (0.65–0.68 nm), being larger than smaller pores of TBBPA-D framework (PDS 0.42–0.59 nm), will find difficultly entering into them. Table 2 lists the molecular size of testing gas/organic vapors and uptake capacities of HBPBA-D and TBBPA-D.

## 4. Conclusions

Micro- and ultra-microporous HBPBA-D and TBBPA-D frameworks were successfully synthesized using six/eight-arm bromophenyl adamantane-based building blocks via a Sonogashira–Hagihara coupling reaction. BET surface areas of the two frameworks were in the region of 395–488 m^2^ g^−1^, with a PSD of 0.92–1.1 nm and 0.42–0.59 nm respectively. Both the frameworks exhibited high thermal stability (stable up to 350 °C prior to decomposition, only 30% mass loss at 800 °C) and good small gas/organic vapor adsorption (8.92–9 wt % of CO_2_ and 1.43–1.63 wt % of CH_4_ at 273 K/1 bar; 104.8–172.5 mg g^−1^ of n-hexane and 144.3–272.3 mg g^−1^ of benzene at 298 K/0.8 bar). The demonstrated high capacities of the synthesized frameworks imply their potential applications in the field of gas/vapor adsorption for environmental protection.

## Figures and Tables

**Table 1:** Porous properties of HBPBA-D and TBBPA-D.

	HBPBA-D	TBBPA-D
S_BET_^1^ (m^2^ g^−1^)	488	395
S_micro_^2^ (m^2^ g^−1^)	243.2	298
V_total_^3^ (cm^3^ g^−1^)	0.29	0.29
V_Micro_^4^ (cm^3^ g^−1^)	0.11	0.13
V_Micro_/V_total_	0.38	0.45
D ^5^ (nm)	0.92–1.1	0.42
D’ ^6^ (nm)	---	0.59

^1^ Surface area calculated from the N_2_ adsorption isotherm using the BET method. ^2^ Microporous surface area calculated using the t-plot method. ^3^ Total pore volume at p/p_0_ = 0.99. ^4^ Micropore volume. ^5^ Pore size distribution (PSD) derived from N_2_ isotherms. ^6^ PSD derived from CO_2_ isotherms.

**Table 2:** Molecular size of gas/organic vapor and uptake capacities of HBPBA-D and TBBPA-D.

Gas/Organic Vapor	Molecular Size	Uptake Capacities	
		HBPBA-D Pore Size: 0.92–1.1 nm	TBBPA-D Pore Size: 0.42–0.59 nm
CO_2_^1^	0.33 nm	9 wt %	8.92 wt %
CH_4_^1^	0.38 nm	1.63 wt %	1.43 wt %
n-hexane ^2^	0.72 nm	172.5 mg g^−1^	104.8 mg g^−1^
Benzene ^2^	0.65–0.68 nm	272.3 mg g^−1^	144.3 mg g^−1^
Selectivity ^3^ (CO_2_/CH_4_)	---	4.1	4.6

^1^ Data were obtained at 273 K/1bar. ^2^ Data were obtained at 298 K/0.8 bar. ^3^ Adsorption selectivity based on the Henry’s law.
